# Comparative Assessment of Schneiderian Membrane Thickness in Smokers and Non-smokers Using Cone-Beam Computed Tomography: An Observational Study

**DOI:** 10.7759/cureus.81481

**Published:** 2025-03-30

**Authors:** Prasad Karande, Jyotismita Thakuria, Akhouri P Sinha, Vikram Karande, Shashwat Asthana, Seema Gupta

**Affiliations:** 1 Department of Oral Pathology and Microbiology, D Y Patil Dental School, Pune, IND; 2 Department of Oral Medicine and Radiology, Government Dental College, Silchar, IND; 3 Department of Oral and Maxillofacial Surgery, Kothiwal Dental College and Research Centre, Moradabad, IND; 4 Department of Oral and Maxillofacial Surgery, D Y Patil Dental School, Pune, IND; 5 Department of Periodontology, Kothiwal Dental College and Research Centre, Moradabad, IND; 6 Department of Orthodontics, Kothiwal Dental College and Research Centre, Moradabad, IND

**Keywords:** augmentation, cone beam computed tomography, schneiderian membrane, smokers, thickness

## Abstract

Introduction: The Schneiderian membrane is crucial in sinus augmentation and implant placement in the posterior maxilla. Its thickness is a key factor in treatment success, as variations may influence surgical outcomes and risk of complications. Smoking has been implicated in mucosal alterations; however, its impact on Schneiderian membrane thickness remains underexplored. This study aimed to compare Schneiderian membrane thickness between smokers and non-smokers using cone-beam computed tomography (CBCT).

Materials and methods: This retrospective study analyzed CBCT scans of 60 individuals divided into two groups: smokers (n = 40) and non-smokers (n = 20). CBCT images were obtained using standardized scanning parameters, and the Schneiderian membrane thickness was measured at multiple sites within the maxillary sinus. Individuals with a history of sinus pathology, maxillary sinus surgery, or systemic diseases affecting mucosal integrity were excluded. Data were analyzed using statistical tests, including independent t-tests, to compare membrane thickness between the two groups.

Results: The mean Schneiderian membrane thickness was significantly greater in smokers than in non-smokers (p < 0.05). Smokers exhibited increased mucosal thickening, particularly in the posterior region of the maxillary sinus. Analysis of the factors influencing sinus mucosal thickness showed no significant association with sex (p = 0.807) or missing teeth status (p = 0.714). However, individuals aged >35 years had significantly higher odds of increased mucosal thickness than those aged <35 years (p = 0.001). In addition, periapical reasons for tooth loss were significantly associated with increased mucosal thickness.

Conclusion: The study findings indicate that smoking is associated with increased Schneiderian membrane thickness, which may have clinical implications for sinus augmentation procedures and implant placement. The altered mucosal characteristics in smokers could contribute to a higher risk of complications such as sinus membrane perforation and sinusitis.

## Introduction

Cone-beam computed tomography (CBCT) has revolutionized the field of dental imaging by offering high-resolution three-dimensional visualization of craniofacial structures. One critical anatomical structure in maxillofacial radiology is the Schneiderian membrane, which lines the maxillary sinus and plays a crucial role in sinus-related procedures, such as sinus lift surgery and dental implant placement [1]. Anatomical variations in the Schneiderian membrane can influence clinical outcomes, particularly in procedures requiring sinus floor elevation. Various factors, including environmental exposure such as smoking, have been implicated in altering the morphology and thickness of the Schneiderian membrane [2]. Smoking is a well-documented risk factor for various systemic and oral health conditions. Inhalation of tobacco smoke introduces a variety of toxic compounds, including nicotine, carbon monoxide, and other carcinogens, which can compromise vascularization, impair mucosal integrity, and induce chronic inflammatory responses. Within the maxillary sinus, these pathophysiological changes may contribute to significant anatomical alterations, including increased mucosal thickening, reduced ciliary function, and a higher prevalence of sinus pathology [3]. Given the widespread prevalence of smoking, understanding its effects on the Schneiderian membrane through CBCT imaging is crucial for optimizing patient management in dental and maxillofacial surgeries.

Several studies have demonstrated that smoking is associated with an increased prevalence of maxillary sinus mucosal thickening, which can be effectively visualized using CBCT [2-4]. Unlike conventional two-dimensional radiographs, CBCT provides superior spatial resolution and allows precise measurement of mucosal changes. By comparing smokers and non-smokers, CBCT imaging can help delineate the extent of smoking-induced changes in the Schneiderian membrane, offering valuable insights for clinicians planning surgical interventions for the posterior maxilla [2,3]. A thicker Schneiderian membrane in smokers may increase the risk of complications such as sinus membrane perforation during sinus augmentation, impaired wound healing, and increased susceptibility to postoperative infections [5]. Schneiderian membrane thickness is crucial in surgery as it influences graft stability and the risk of sinus perforation, which can compromise implant success. Thicker membranes may provide better support, while thinner ones increase perforation risk. Smoking contributes to mucosal alterations by inducing inflammation, reducing vascularity, and impairing healing, all of which can compromise surgical outcomes.

Additionally, smoking has been linked to a higher incidence of maxillary sinus pathologies, including sinusitis and polyp formation [5]. Chronic exposure to tobacco smoke may lead to impaired mucociliary clearance and persistent inflammatory changes in the sinus epithelium, contributing to pathological alterations that can be detected radiographically [6]. The ability of CBCT to detect these variations enables clinicians to assess preoperative risks more effectively and to tailor surgical approaches accordingly. For instance, in smokers with significant mucosal thickening, alternative sinus augmentation techniques or preoperative management strategies may be required to minimize complications [2].

Despite the growing body of evidence linking smoking to Schneiderian membrane alterations, there remains a need for more comprehensive research using CBCT to establish definitive correlations. Most existing studies rely on traditional radiographic techniques that lack the precision required for detailed morphometric analyses. CBCT-based evaluations can provide a clearer understanding of the impact of smoking on sinus anatomy, thereby refining clinical decision making and improving surgical outcomes.

This study aimed to compare the anatomical variations in the Schneiderian membrane between smokers and non-smokers using CBCT imaging. Consequently, the objectives of the present investigation were to (1) examine the correlation between smoking behaviors, including smoking classification (current, former, and non-smokers), smoking intensity (daily cigarette consumption), smoking duration (years of smoking), cumulative exposure (pack-years), and Schneiderian membrane thickness within the maxillary sinus using CBCT imaging; and (2) evaluate the impact of smoking-associated variables and dental health status on the mucosal thickening observed in various anatomical regions of the maxillary sinus.

## Materials and methods

Study design and setting

This study was conducted as a retrospective observational study on the CBCT records of patients at the Department of Oral and Maxillofacial Surgery, Kothiwal Dental College and Research Centre, Moradabad, India. The study spanned a period of two years from July 2024 to March 2022 and was performed following ethical approval from the institutional ethical review board (KDCRC/IERB/08/2024/SH21). As a routine protocol of the department, written informed consent was obtained from the patients to use their records for study purposes and to maintain their confidentiality. This study was conducted in accordance with the principles of the Declaration of Helsinki.

Sample size estimation

Sample size estimation was performed using G*Power software (version 3.6.9; Heinrich-Heine-Universität Düsseldorf, Düsseldorf, Germany) to achieve a statistical power of 80%, with an alpha error of 5%. This calculation was based on an effect size of 0.46, as reported in a previous study by Hung et al. [5], who investigated the sinus mucosal lining thickness in smokers and non-smokers. These parameters were analyzed using an independent t-test. The a priori computation yielded a total sample size of 60, distributed in a ratio of (1:2) in two groups with 20 non-smokers and 40 smokers, ensuring robust statistical validity. ​The unequal group sizes- 40 smokers versus 20 non-smokers - were chosen to reflect the higher prevalence of smoking within the study's target population. This approach enhances the study's external validity by ensuring the sample more accurately represents real-world demographics. Additionally, the larger smoker group increases the statistical power to detect differences related to smoking behaviors. While equal group sizes are often preferred for statistical comparisons, in observational studies, proportional sampling can provide a more accurate reflection of population characteristics.

Eligibility criteria

The study included adults aged ≥ 18 years who underwent high-quality CBCT imaging for dental assessment, implant planning, or sinus evaluation. Sixty CBCT records were categorized into two groups: smokers (n = 40) and non-smokers (n = 20). The inclusion criteria were that patients had no history of maxillary sinus surgery or trauma and had at least one posterior maxillary tooth or a healed edentulous ridge to allow for the assessment of dental status. Patients with systemic conditions that could influence sinus mucosal health, such as uncontrolled diabetes (defined by a glycated haemoglobin as HbA1c level exceeding 10%) or immunosuppressive disorders, were also excluded. Other exclusion criteria included acute or chronic sinus infections within the past three months, prior radiation therapy to the head and neck, long-term use of medications affecting mucosal health (such as corticosteroids or bisphosphonates), and the presence of maxillary sinus cysts or tumors unrelated to smoking.

Methodology

Patient demographics, history of tobacco use (including current, former, or never smokers), daily tobacco consumption, and smoking duration at the time of imaging were extracted from the patients' medical records. CBCT scans were acquired using Carestream New Generation CBCT apparatus (Carestream Dental, Atlanta, GA) in accordance with a standardized protocol (operating at a voltage of 120 kV, a current of 80 mA, seven-second scan time, a field of view (FOV) measuring 10 × 10 cms, a resolution of 0.2 mm^3^ voxels, and 1 mm slice thickness). To achieve consistent head orientation, the subjects were stabilized by the application of a head stabilizer. All scans were taken with the patients positioned upright, ensuring that the maxillary occlusal plane was parallel to the floor. The participants were instructed to remain still and breathe normally to avoid motion artifacts. Image reconstruction was performed in the coronal, axial, and sagittal planes to allow detailed visualization of the maxillary sinus.

**Figure 1:**
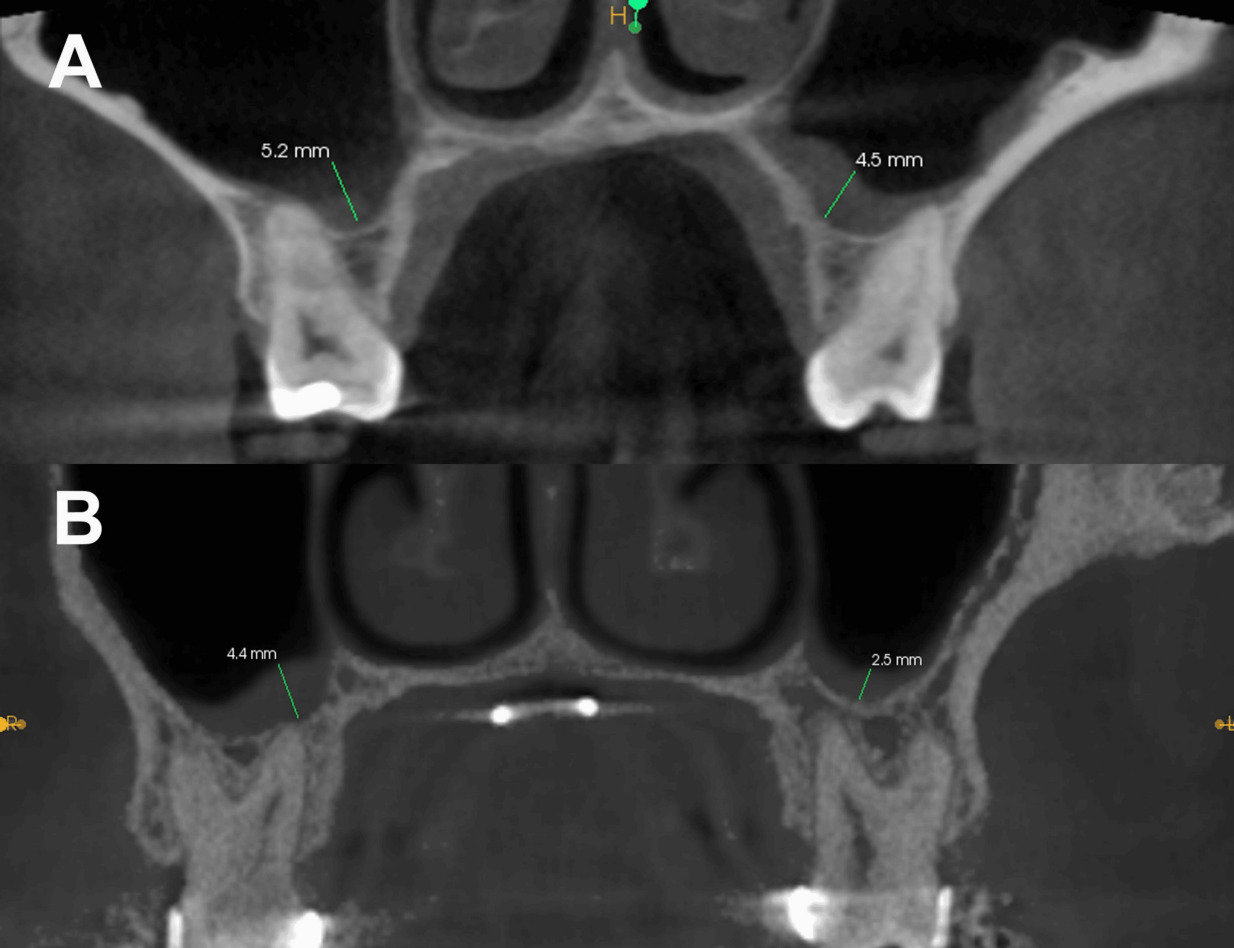
Maxillary sinus floor lining thickness in mm A: Smokers; B: Non-smokers This figure is taken from a CBCT scan of a patient included in the study.

Schneiderian membrane thickness was measured using dedicated CBCT software following a standardized approach. Measurements of mucosal thickness were performed on cross-sectional images to ensure accuracy and avoid oblique distortions.​ Three predetermined locations within the maxillary sinus - anterior (in the region of the second premolar), middle (in the region of the first permanent molar), and posterior (in the region of the second permanent molar) - were selected for measurements. A perpendicular line was drawn from the sinus floor to the outermost point of the mucosa at each site, and measurements were performed bilaterally (Figure 1).

Mucosal thickening was considered normal if it was less than 2 mm [7]. Sinus membrane thickness was compared among smoking groups, with additional subgroup analyses based on smoking intensity (daily cigarette consumption), duration (years of smoking), and cumulative exposure (pack-years). To ensure measurement reliability, intra- and inter-examiner agreements were assessed. Two independent calibrated examiners, who were blinded to the group allocation, performed the measurements and repeated the measurements on a random selection of 10 CBCT scans. To evaluate intra- and inter-examiner reliability, both examiners repeated the measurements after a two-week interval. The intraclass correlation coefficient (ICC) was calculated, and values above 0.80 were considered indicative of excellent reliability.

Statistical analysis

Data analysis was performed using the SPSS software Statistical Product and Service Solutions (SPSS, version 23.0; IBM SPSS Statistics for Windows, Armonk, NY). All data were tested for normality using the Kolmogorov-Smirnov test and were found to be normally distributed. Categorical data are presented as frequencies and percentages, while continuous data are presented as means and standard deviations. Chi-square analysis was performed to determine the association of categorical variables with mucosal thickness, and an independent t-test was used to compare the mean mucosal thickness between non-smokers and smokers. In addition to this linear regression, an odds ratio (OR) was performed for factorial analysis with a significance threshold set at p < 0.05. In the regression model, the independent variables included smoking status (current, former, non-smoker), smoking intensity (daily cigarette consumption), smoking duration (years of smoking), and cumulative exposure (pack-years). The model was adjusted for potential confounders, such as age, sex, and missing teeth status, to ensure accurate assessment of the relationship between smoking variables and Schneiderian membrane thickness.​ Regarding multiple subgroup analyses, appropriate correction methods, such as the Bonferroni correction, were applied to control for type I errors.

## Results

ICC values of 0.85 for inter-examiner and 0.89 for intra-examiner reliability showed excellent reliability and reproducibility. The distribution of the study population showed no statistically significant differences in sex (p = 0.099) and age group (p = 1) between smokers and non-smokers. Similarly, missing tooth (p = 0.409) and the reason for tooth absence (p = 0.551) did not show significant associations with smoking status. However, mucosal thickening was significantly associated with smoking, indicating a notable difference between smokers and non-smokers in this parameter (Table 1).

**Table 1:** Distribution of the study population *p-value < 0.05: significant, p-value was calculated using the chi-square test of association, and data are presented in the form of n (%).

Parameters	Category	Non-smokers n (%)	Smokers n (%)	Chi value	p-value
Sex	Male	12 (20%)	32 (54%)	2.73	0.099
	Female	8 (13%)	8 (13%)		
Age group	<35 years	9 (15%)	18 (30%)	0	1
	>35 years	11 (18%)	22 (37%)		
Mucosal thickening	<2 mm	13 (22%)	8 (13%)	11.87	0.001*
	>2 mm	7 (12%)	32 (53%)		
Missing tooth	No	16 (27%)	28 (47%)	0.68	0.409
	Yes	4 (7%)	12 (20%)		
Reason for tooth absence	Periapical	2 (13%)	8 (50%)	0.36	0.551
	Periodontal	2 (13%)	4 (25%)		

As no statistically significant differences were noted in mucosal thickening on the right and left sides, the values were averaged for further analysis. The comparison of mucosal thickening across different variables showed significant associations with age (p = 0.001), duration in years (p = 0.001), cumulative exposure (p = 0.001), and mucosal thickening measurement (p = 0.001), indicating that individuals with mucosal thickening >2 mm had higher values for these factors. However, smoking frequency per day did not show a significant difference between the groups (p = 0.492). These findings suggest that older age, longer smoking duration, and higher cumulative exposure are strongly associated with increased mucosal thickening, while daily smoking frequency alone may not have a direct impact (Table 2).

**Table 2:** Comparison of mucosal thickening (mm) in different variables *p-value < 0.05: significant, p-value was calculated using the independent t-test, and data are presented in the form of mean and standard deviation (SD).

Parameters	>2 mm	<2 mm	t stats	p-value
	Mean±SD	Mean±SD		
Age (years)	41.41±8.36	29.57±6.24	5.68	0.001*
Duration (years)	13.44±6.78	2.88±1.36	4.34	0.001*
Frequency (per day)	4.56±1.24	4.25±0.46	0.69	0.492
Cumulative exposure	3.12±1.85	0.63±0.37	3.77	0.001*
Mucosal thickening (mm)	4.7±1.44	1.44±0.39	10.14	0.001*

The comparison of mean mucosal thickening revealed no statistically significant differences based on sex (p = 0.093) or missing teeth status (p = 0.442). However, smokers exhibited significantly higher mucosal thickening than non-smokers (p = 0.001). Additionally, periapical reasons for tooth loss were associated with greater mucosal thickening compared to periodontal causes (p = 0.045). A significant difference was also observed in cumulative smoking exposure, where individuals with smoking exposure had higher mucosal thickening (p = 0.001). These findings suggest that smoking, periapical-related tooth loss, and cumulative smoking exposure may contribute to increased mucosal thickening (Table 3).

**Table 3:** Comparison of mean mucosal thickening for the study variables *p-value < 0.05: significant, p-value was calculated using the independent t-test, and data are presented in the form of mean and standard deviation (SD).

Parameters	Category	n (%)	Mean±SD	t stat	p-value
Sex	Male	44 (73.33%)	3.82±2.1	1.71	0.093
	Female	16 (26.67%)	2.86±1.4		
Smokers	Yes	40 (66.67%)	4.42±1.8	6.08	0.001*
	No	20 (33.33%)	1.85±0.9		
Missing teeth	No	44 (73.33%)	3.44±2.0	-0.77	0.442
	Yes	16 (26.67%)	3.89±2.0		
Reason of missing teeth	Periapical	10 (16.95%)	4.27±2.4	1.98	0.045*
	Periodontal	6 (10.17%)	3.25±1.2		

The analysis of factors influencing sinus mucosal thickness (>2 mm) showed no significant association with sex (p = 0.807, OR = 0.86) or missing teeth status (p = 0.714, OR = 0.8). However, individuals aged >35 years had significantly higher odds of increased mucosal thickness compared to those <35 years (p = 0.001, OR = 12.32). Smoking was found to be a significant protective factor, with smokers showing lower odds of mucosal thickening (p = 0.001, OR = 0.13). Additionally, periapical reasons for tooth loss were significantly associated with increased mucosal thickness compared to periodontal causes (p = 0.003, OR = 6.12). These findings suggest that age >35 years and periapical-related tooth loss may contribute to increased sinus mucosal thickness, while smoking appears to have an inverse association (Table 4).

**Table 4:** Factors influencing sinus mucosal thickness (>2 mm rate: odds ratio as OR) *p-value < 0.05: significant, p-value was calculated using the independent t-test, and data are presented in the form of n (%).

Parameter	Category	n (%)	p-value	Odds ratio
Sex	Male	29 (74%)	0.807	0.86
	Female	10 (26%)		
Age group	>35 years	29 (74%)	0.001*	12.32
	<35 years	10 (26%)		
Smoking	Yes	32 (82%)	0.001*	0.13
	No	7 (18%)		
Reason	Periapical	7 (18%)	0.003*	6.12
	Periodontal	4 (11%)		
Missing tooth	No	28 (72%)	0.714	0.8
	Yes	11 (28%)		

The linear regression analysis for mucosal lining thickness showed that age (p = 0.359) and smoking frequency per day (p = 0.191) were not significantly associated with mucosal thickness. However, duration in years (p = 0.036) and cumulative exposure (p = 0.019) were significant predictors, indicating that longer exposure duration and higher cumulative exposure contribute to increased mucosal thickness. These findings suggest that, while age and daily smoking frequency may not have a direct impact, the overall duration and cumulative exposure to smoking significantly influence mucosal thickening (Table 5).

**Table 5:** Linear regression analysis for mucosal lining thickness with factors *p-value < 0.05: significant.

Model	Coefficients Beta	T stats	p-value	95% confidence interval for B	
				Lower bound	Upper bound
Age (years)	0.23	0.93	0.359	-0.06	0.15
Duration (years)	0.25	2.48	0.036*	0.31	0.19
Frequency (per day)	0.35	-1.33	0.191	-1.38	0.29
Cumulative exposure	0.8	3.33	0.019*	0.38	1.84

## Discussion

The present study aimed to evaluate the influence of smoking on Schneiderian membrane thickness by using CBCT. These results demonstrated a significant association between smoking and increased maxillary sinus mucosal thickening. Specifically, smoking duration and cumulative exposure (pack-years) emerged as significant predictors of mucosal thickness, whereas daily smoking frequency did not show a direct impact. These findings align with previous studies that have reported an increased prevalence of sinus mucosal thickening among smokers compared to non-smokers, thereby reinforcing the hypothesis that chronic exposure to tobacco smoke contributes to anatomical alterations in the maxillary sinus mucosa [1-3].

Previous studies have consistently documented detrimental effects of smoking on sinus health, including increased mucosal thickening, reduced mucociliary clearance, and higher susceptibility to sinus infections [2-4]. Smoking introduces various toxins, including nicotine, carbon monoxide, and other carcinogens, which impair vascularization and induce chronic inflammation, leading to sinus mucosal changes [5]. The results of our study are consistent with those of Reh et al. [6], who found that smoking is associated with increased maxillary sinus membrane thickening. Similarly, Shanbhag et al. [8] reported that smokers exhibited a significantly greater prevalence of mucosal thickening than non-smokers, with a higher incidence of sinus pathology.

Our study further strengthens the evidence that CBCT imaging is a valuable tool for assessing smoking-induced sinus mucosal alterations. Conventional two-dimensional radiographs have limitations in accurately assessing the thickness and morphology of the Schneiderian membrane. In contrast, CBCT provides high-resolution three-dimensional visualization, allowing for precise measurement of mucosal thickness at different anatomical sites [9]. The ability to objectively quantify these changes has important clinical implications for surgical planning in procedures such as sinus augmentation and dental implant placement.

A key finding of this study was the strong correlation between smoking duration and cumulative exposure to Schneiderian membrane thickness. While the daily smoking frequency did not show a significant impact, individuals with prolonged smoking histories exhibited greater mucosal thickening. This suggests that chronic exposure to tobacco smoke over time is a more critical factor for sinus alterations than the absolute number of cigarettes smoked per day. The principal ostia of both maxillary sinuses are situated within the middle meatus of the nasal cavity; consequently, the maxillary sinus may exhibit increased vulnerability to the impact of inhaled smoke owing to its direct anatomical linkage with the middle meatus of the nasal cavity. Similar findings have been reported in previous studies [5,10], which found that cumulative smoking exposure, rather than smoking intensity, was a significant predictor of sinus mucosal thickening. A study by Pleasants et al. [11] encompassed a range of indicators (intensity, duration, and cumulative pack-years of tobacco smoking) in an analytical framework. The findings indicated that individuals exhibiting heightened intensity, extended duration, and increased pack-years of smoking were predisposed to the presence of mucosal thickening in the ethmoid sinus. Although no statistically significant variances were observed in the cumulative pack-years, individuals with elevated intensity and duration of smoking demonstrated an increased likelihood of experiencing mucosal thickening of the maxillary sinus.

The observed increase in mucosal thickness in smokers can be attributed to several pathophysiological mechanisms. Chronic exposure to cigarette smoke leads to persistent inflammation and vascular congestion in the sinus mucosa, resulting in epithelial hyperplasia and submucosal edema [12]. Additionally, smoking-induced impairment of mucociliary clearance disrupts normal sinus drainage, predisposing individuals to sinus infections and further mucosal thickening [4]. This finding has also been observed in passive smokers [13]. A combination of these factors contributes to a cycle of chronic sinus inflammation and altered mucosal morphology, which can be effectively visualized using CBCT imaging.

Our study also revealed that individuals above the age of 35 years had significantly higher odds of increased mucosal thickness than younger individuals. This finding is consistent with previous research by Lu et al. [14], which indicated that age-related physiological changes, combined with prolonged exposure to environmental and lifestyle factors, such as smoking, contribute to sinus mucosal alterations. Although aging alone is associated with changes in the respiratory epithelium, the cumulative impact of smoking exacerbates these effects. Prolonged inflammatory insult from tobacco exposure over decades may lead to more pronounced mucosal hypertrophy in older individuals. Our study further indicated that periapical infections were associated with increased mucosal thickness, which aligns with a previous study by Aksoy et al. [15], who reported increased mucosal thickening in cases of periapical infections and missing teeth. The prevalence rate of mucosal thickening in cases of odontogenic infections has been reported to be 37-42% in previous studies [16,17]. The severity of this thickening varies, influenced by factors such as the virulence of the infecting bacteria and the host's immune response. For instance, teeth with periodontal bone loss are 2.2 times more likely to be associated with mucosal thickening than those with periapical or combined lesions [18]. Variability in host immune responses and differences in infection severity contribute to why some periapical infections lead to significant mucosal thickening while others do not [15].

Goller-Bulut et al. [18] indicated that, within a cohort of teeth exhibiting apical periodontitis, the first molar emerged as the most significantly impacted tooth, followed by the first premolars. Shanbhag et al. [19] documented that teeth presenting periapical lesions predominantly included the first and second molars. This finding is more evident in older individuals, as they exhibit a heightened vulnerability to oral health disorders, including periodontal disease, apical abscesses, edentulism, and various other pathological states associated with aging, which consequently elevates the incidence of maxillary sinusitis.

Following pulp necrosis, potent bacterial virulence factors, including collagenase, lysosomal enzymes, and toxins, facilitate bacterial invasion and subsequent degradation of the periapical bone [20]. Consequently, infections and their resultant by-products emanating from dental sources may disseminate to the maxillary sinuses, thereby posing a potential risk of mucosal irritation within the sinus. As bacteria infiltrate the maxillary sinus, the symptoms are expected to escalate progressively in conjunction with the development of sinusitis. Upon eradication of tooth-associated infection, the literature documents a swift resolution of sinusitis symptoms [21].

​Smoking cessation before surgery is recommended to reduce postoperative complications, but achieving patient adherence is challenging due to nicotine addiction. The optimal timing for preoperative cessation is debated; some studies suggest that short-term cessation (four to six weeks) may improve wound healing and reduce pulmonary complications, though its direct effect on mucosal thickening is less clear [22]. Pharmacological interventions, such as corticosteroids and nasal decongestants, can reduce mucosal inflammation before surgery, potentially enhancing surgical outcomes [23]. Clinicians often use the pack-years metric (packs smoked per day multiplied by years smoked) to quantify cumulative smoking exposure. Specific thresholds for risk stratification vary, but higher pack-years generally correlate with increased surgical risks [11]. Mucosal thickening is also relevant in other clinical scenarios, such as sinusitis management and orthodontics, indicating that these findings have implications across various medical specialties.

The study's finding that daily smoking frequency was not significantly associated with mucosal thickening may be due to variability in patient reporting or the greater impact of cumulative exposure over time. Older age and periapical tooth loss could serve as independent risk factors for mucosal thickening or may result from smoking-related damage, necessitating further research to clarify their interactions. While preoperative CBCT assessments are valuable for surgical planning, practical constraints such as cost and availability suggest that routine use may not be feasible; reserving CBCT for high-risk cases could be a more balanced approach.

Clinical implications of the study

The findings of this study have several important clinical implications, particularly for dental and maxillofacial surgical procedures involving the maxillary sinuses. Sinus floor elevation procedures, which are commonly performed to facilitate implant placement in the posterior maxilla, require careful evaluation of the Schneiderian membrane. Excessive mucosal thickening increases the risk of membrane perforation, which can lead to postoperative complications, such as sinus infections and implant failure [2,3]. Given the association between smoking and increased mucosal thickening, clinicians should consider pre-operative screening for sinus membrane alterations in smokers. CBCT imaging provides an effective means of assessing mucosal changes, allowing individualized treatment planning. In cases where significant mucosal thickening is detected, alternative surgical approaches, such as the use of a lateral window technique or preoperative management strategies (e.g., smoking cessation counselling or pharmacological interventions to reduce mucosal inflammation), may be warranted to minimize surgical risks. Additionally, our study highlights the importance of considering cumulative smoking exposure rather than smoking frequency when evaluating patient risk profiles. Patients with long-term smoking histories should be counselled about the potential impact of smoking on sinus health and the increased risk of complications of maxillary sinus procedures. Smoking cessation before surgery may help improve sinus mucosal integrity and reduce the risk of postoperative complications.

Limitations and future directions

Despite the valuable insights provided by this study, it has some limitations that warrant consideration. First, the retrospective nature of the study may have introduced selection bias, as only patients with existing CBCT records were included. Second, the study relied on self-reported smoking history, which may be subject to recall bias and underreporting. Future studies incorporating objective biomarkers of tobacco exposure (e.g., as cotinine levels) could provide more accurate assessments of smoking behavior and its impact on sinus anatomy. Another limitation was the relatively small sample size. Although statistical power was sufficient to detect significant associations, larger-scale studies with diverse populations would be beneficial to further validate these findings. Additionally, longitudinal studies tracking changes in Schneiderian membrane thickness over time among smokers and non-smokers could provide deeper insights into the progression of smoking-induced sinus alterations. Finally, while this study focused primarily on smoking as a risk factor for mucosal thickening, other environmental and systemic factors, such as air pollution, allergies, and chronic rhinosinusitis, could also influence sinus health. ​Our study did not investigate potential non-linear relationships between smoking intensity and Schneiderian membrane thickness, such as threshold effects at higher smoking doses. Future research employing advanced statistical methods is recommended to explore these complex associations. Future research should also explore the interplay between these variables and smoking-related sinus changes. Environmental factors, such as air pollution, allergies, and chronic rhinosinusitis, could confound the relationship between smoking and mucosal thickening. Future research should control for these variables to isolate the specific effects of smoking, as their influence may be substantial and not necessarily minor compared to smoking.

## Conclusions

This study highlights the significant impact of smoking on Schneiderian membrane thickness as evaluated using CBCT imaging. Our findings demonstrate that smokers exhibit greater mucosal thickening than non-smokers, with cumulative smoking exposure and longer smoking duration emerging as the key predictors of increased thickness. Notably, while daily smoking frequency did not show a significant association, a prolonged smoking history was strongly correlated with maxillary sinus mucosal alterations. Additionally, factors such as older age and periapical tooth loss are associated with increased mucosal thickness. These findings underscore the importance of preoperative CBCT assessment in smokers to optimize treatment planning for sinus-related dental procedures, particularly in implantology and sinus augmentation surgeries.
